# New Insight Into Avian Papillomavirus Ecology and Evolution From Characterization of Novel Wild Bird Papillomaviruses

**DOI:** 10.3389/fmicb.2019.00701

**Published:** 2019-04-12

**Authors:** Marta Canuti, Hannah J. Munro, Gregory J. Robertson, Ashley N. K. Kroyer, Sheena Roul, Davor Ojkic, Hugh G. Whitney, Andrew S. Lang

**Affiliations:** ^1^Department of Biology, Memorial University of Newfoundland, St. John’s, NL, Canada; ^2^Wildlife Research Division, Environment and Climate Change Canada, Mount Pearl, NL, Canada; ^3^Animal Health Laboratory, University of Guelph, Guelph, ON, Canada

**Keywords:** papillomavirus, avian papillomavirus, virus discovery, virus evolution, viral ecology, molecular epidemiology

## Abstract

Viruses in the family *Papillomaviridae* have circular dsDNA genomes of approximately 5.7–8.6 kb that are packaged within non-enveloped, icosahedral capsids. The known papillomavirus (PV) representatives infect vertebrates, and there are currently more than 130 recognized PV species in more than 50 genera. We identified 12 novel avian papillomavirus (APV) types in wild birds that could represent five distinct species and two genera. Viruses were detected in paired oropharyngeal/cloacal swabs collected from six bird species, increasing the number of avian species known to harbor PVs by 40%. A new duck PV (DuPV-3) was found in mallard and American black duck (27.6% estimated prevalence) that was monophyletic with other known DuPVs. A single viral type was identified in Atlantic puffin (PuPV-1, 9.8% estimated prevalence), while a higher genetic diversity was found in other Charadriiformes. Specifically, three types [gull PV-1 (GuPV-1), -2, and -3] were identified in two gull species (estimated prevalence of 17% and 2.6% in American herring and great black-backed gull, respectively), and seven types [kittiwake PV-1 (KiPV-1) through -7] were found in black-legged kittiwake (81.3% estimated prevalence). Significantly higher DuPV-3 circulation was observed in spring compared to fall and in adults compared to juveniles. The studied host species’ tendencies to be in crowded environments likely affect infection rates and their migratory behaviors could explain the high viral diversity, illustrating how host behavior can influence viral ecology and distribution. For DuPV-3, GuPV-1, PuPV-1, and KiPV-2, we obtained the complete genomic sequences, which showed the same organization as other known APVs. Phylogenetic analyses showed evidence for virus–host co-divergence at the host taxonomic levels of family, order, and inter-order, but we also observed that host-specificity constraints are relaxed among highly related hosts as we found cross-species transmission within ducks and within gulls. Furthermore, the phylogeny of viruses infecting the Charadriiformes did not match the host phylogeny and gull viruses formed distinct monophyletic clades with kittiwake viruses, possibly reflecting past host-switching events. Considering the vast PV genotype diversity in other hosts and the large number of bird species, many more APVs likely remain to be discovered.

## Introduction

Papillomaviruses (PVs) are small, non-enveloped, icosahedral viruses of vertebrates with a circular dsDNA genome of approximately 5.7–8.6 kb. They belong to the family *Papillomaviridae*, which currently contains more than 130 recognized species in more than 50 genera. Most PVs are contained within the subfamily *Firstpapillomavirinae* and are associated with amniote hosts (reptiles, birds, and mammals), while the subfamily *Secondpapillomavirinae* contains a single viral species that has been identified in a fish (Van Doorslaer et al., 2018). Their genomes contain several open-reading frames (ORFs), all located on the same DNA strand, encoding the structural or late proteins (L1 and L2) and several nonstructural or early proteins (E1–E9), and a non-coding regulatory region known as the long control region (LCR). Some viruses also possess a second non-coding region. Although the genomic structure is similar across different species, the number of encoded E proteins is variable and only the core ORFs E1, E2, L2, and L1 are present in all PV genomes characterized to date (Rector and Van Ranst, 2013).

Papillomaviruses primarily infect mucosal (mucocutaneous PVs) and keratinized (cutaneous PVs) epithelia and are commonly found on healthy skin (Antonsson et al., 2003; Antonsson and McMillan, 2006). Although most infections are subclinical, PV infection can be productive and cause benign epithelial proliferation with the development of typical skin lesions (warts) that commonly undergo spontaneous regression, but which may occasionally progress to cancers, such as skin squamous cell carcinomas, gastrointestinal, or urinary cancers (Nicholls and Stanley, 2000; Campo, 2003; Cubie, 2013).

Most of the currently known PVs infect humans, but with modern improved sequencing technologies and virus discovery methods the number of known animal PVs is rapidly increasing, revealing a possibly comparable genotype diversity in other animals (Rector and Van Ranst, 2013; Bravo and Félez-Sánchez, 2015). Originally, PVs were considered to be strictly host species-specific, with only rare cases of cross-species transmission, indicative of a long virus–host association and a subsequent tendency of viruses to codiverge with their hosts (Shah et al., 2010). However, this paradigm is vacillating (Gottschling et al., 2007). In fact, although virus–host coevolution and intra-host duplication are still considered the principal driving forces for PV evolution, other evolutionary forces, such as host-switching, seem to be involved, and a broad host range may be more common than anticipated (Shah et al., 2010; Bravo and Félez-Sánchez, 2015). For example, several distantly related PVs have been observed in bats (García-Pérez et al., 2014) and some viruses are able to cross species borders, such as some bovine PV types that can also infect various other herbivores, equids, and cats (Chambers et al., 2003; Costa et al., 2017), with species-specificity being less strict among genetically closely related hosts (Gottschling et al., 2007; Chen et al., 2009).

Considering the vast viral diversity known for other vertebrates, our knowledge about avian papillomaviruses (APVs) is still very limited. In fact, to our knowledge, only eight APV species have been described so far in seven bird species in six orders, and clinical data are only available for some of these. The first evidence of viral involvement in skin lesions on bird legs goes back to the early 1970s, when Lina et al. (1973) reported evidence from electron microscopy for the presence of PVs in squamous papilloma tissues collected from chaffinches (*Fringilla coelebs*). Since then, the presence of this virus in cutaneous lesions of chaffinches has been confirmed several times in different European countries (Literák et al., 2012; Truchado et al., 2018b).

Besides the *F. coelebs* PV (FcPV-1), the *Psittacus erithacus timneh* PV (PePV-1) and the *Fulmarus glacialis* PV (FgPV-1) have been identified in association with cutaneous tumors of the African gray parrot and the northern fulmar, respectively (Tachezy et al., 2002; Terai et al., 2002; Gaynor et al., 2015). Furthermore, a PV was recently identified in cutaneous lesions of a griffon vulture (*Gyps fulvus*) (Di Francesco et al., 2019) and the *Francolinus leucoscepus* PV (FlPV-1) was identified on the healthy skin of a yellow-necked francolin (Van Doorslaer et al., 2009). There is also evidence for the existence of mucocutaneous APVs in birds: the *Serinus canaria* PV (ScPV-1) was detected in the oral cavity of canaries (Truchado et al., 2018a) and three other APV species were identified in cloacal swabs or fecal content of the Adélie penguin [*Pygoscelis adeliae* PV-1 (PaPV-1) and -2] and in mallard [*Anas platyrhynchos*; duck PV (DuPV)] (Varsani et al., 2014; Fawaz et al., 2016; Van Doorslaer et al., 2017b; Williams et al., 2018). Two independent studies that analyzed samples from India and Sweden reported identification of the same PV (DuPV-1) in mallard, with a second variant, DuPV-2, observed in the Swedish study only (Fawaz et al., 2016; Williams et al., 2018).

We report here the full genomic sequence and molecular characterization of four novel APVs we discovered in four different bird species: Atlantic puffin (*Fratercula arctica*), American herring gull (*Larus smithsonianus*), mallard, and black-legged kittiwake (*Rissa tridactyla*). We also report partial sequences of an additional eight potentially novel PVs identified in herring gull, great black-backed gull (*Larus marinus*), and black-legged kittiwake. Furthermore, we estimated the prevalence and evaluated the molecular epidemiology of these viruses in different bird populations and analyzed their evolutionary relationships in the context of all currently known APVs.

## Materials and Methods

### Sample Collection

This study investigated 459 samples collected from 401 individual birds between 2012 and 2018 in Newfoundland and Labrador, Canada, during past and current avian influenza A virus (AIV) surveillance studies (Huang et al., 2013; Wille et al., 2014). This study was carried out in accordance with the recommendations of the Canadian Council on Animal Care. The protocol was approved by the Memorial University Institutional Animal Care Committee (approved protocols 11-01-AL, 12-01-AL, 13-01-AL, 14-01-AL, and 17-05-AL). Wild birds were captured under federal authority (Canadian Wildlife Service Migratory Bird Banding Permit 10559). Samples (all AIV-negative) included oropharyngeal and cloacal swabs collected from birds in three families: Alcidae [51 Atlantic puffin, 41 common murre (*Uria aalge*), 2 thick-billed murre (*Uria lomvia*), 30 razorbill (*Alca torda*)], Laridae [94 American herring gull, 38 great black-backed gull, 4 Iceland gull (*Larus glaucoides*), 9 ring-billed gull (*Larus delawarensis*), 16 black-legged kittiwake], and Anatidae [102 American black duck (*Anas rubripes*), 10 mallard, 4 American black duck × mallard hybrids].

Samples from Atlantic puffin, common murre, and razorbill were collected at the Gannet Islands and Witless Bay Ecological Reserves, Newfoundland and Labrador, which are two major seabird colonies in eastern Canada. Samples from thick-billed murre were collected only at the Gannet Islands and those from black-legged kittiwakes were collected only in Witless Bay. Gull samples were collected at a regional landfill (herring and great black-backed gull) or nearby at Quidi Vidi Lake (Iceland and ring-billed gull), both within the city of St John’s, Newfoundland and Labrador, Canada, while ducks were sampled at three different ponds within St John’s. Date, location of sampling, and age and sex (only for ducks) of swabbed birds were recorded. Most sampled birds were adults (≥1-year-old, *N* = 312, including all Alcidae, all kittiwakes, 91 gulls, and 79 ducks), while samples from juveniles (<1-year-old) were available only for ducks (*N* = 35) and gulls (American herring, great black-backed, and ring-billed gulls, *N* = 54).

In most cases both oropharyngeal and cloacal swabs (polyester swabs, Starplex Scientific) were submerged together into 3 ml viral transport medium (Starswab Multitrans System, Starplex Scientific), while for samples collected in 2018 (*N* = 12, all from ducks) the two swabs were preserved separately. Samples were kept frozen until processing.

### Complete Genome Sequencing

Small genomic fragments of different APVs, previously identified with the ViDiT-CACTUS virus discovery method (Verhoeven et al., 2018), were used as templates for primer design for complete genome sequencing, which was achieved by a combination of specific PCRs and genome walking, followed by Sanger sequencing. All primers used in this study are available upon request.

The ViDiT-based genome walking (ViDiWa) method was used to amplify unknown genomic regions flanking known sequences. During a first amplification round a virus-specific primer was used in combination with a tailed random primer (ViDiT_AXXN) to amplify the target genomic region, while a secondary amplification was performed to increase the output using a nested virus-specific primer and a primer annealing to the tail of the random primer (ViDIT_AXX). In detail, nucleic acids (NAs) were isolated from 200 μl of thoroughly vortexed transport medium sample with the DNeasy Blood and Tissue kit (Qiagen) and 2.5 μl isolated NAs was used as input in a 25 μl reaction mix containing 0.2 μM specific primer, 0.8 μM random primer, and 1× DreamTaq Green PCR Master Mix (Thermo Fisher Scientific). The amplification was performed according to the following thermoprofile: 5 min at 95°C followed by 15 cycles at 95°C for 30 s, 55°C for 30 s, and 72°C for 2 min (to allow for specific DNA enrichment), followed by 1 cycle at 95°C for 30 s, 26°C for 2 min, and 72°C for 2 min (to allow random amplification), and 30 cycles at 95°C for 30 s, 55°C for 30 s, and 72°C for 2 min with a final 7-min incubation at 72°C. Following the first amplification, PCR clean-up was performed with Agencourt AMPure XP beads (0.85:1 v:v) (Beckman Coulter) and 5 μl of the purified DNA was used for the second amplification round in a 25 μl reaction mix containing 0.2 μM primers and 1× DreamTaq Green PCR Master Mix. The second amplification was run for 5 min at 95°C followed by three cycles of 95°C for 30 s, 48°C for 30 s, 72°C for 2 min, and 35 cycles of 95°C for 30 s, 55°C for 30 s, 72°C for 2 min, and completed with 7 min at 72°C. ViDiT primer sequences and PCR clean-up procedures were the same as described previously (Verhoeven et al., 2018).

### Screening and L1 Sequencing

Swabs were thoroughly vortexed within the transport medium and NAs were isolated with TRIzol^TM^ Reagent (Thermo Fisher Scientific) for the American black duck samples collected in 2015, the QIAamp DNA Mini Kit for the 2018 samples, or the MagMAX-96 Viral RNA Isolation Kit (Thermo Fisher Scientific) for all other samples.

Primers specific to each identified virus were designed and sample screening was performed by hemi-nested PCRs with primers listed in Table 1. Screening involved the amplification of 268-nt, 138-nt, and 267-nt fragments of the L1 ORF of the puffin, gull and kittiwake, and duck viruses, respectively. PCR-positive samples were confirmed by sequencing and a subset of samples was selected for further analyses. In particular, the entire L1 ORF was sequenced for several viruses and the sequence used for molecular-epidemiological investigations (primers available upon request).

**Table 1:** Primers (5’–3’) used for APV screening.

Primer	Sequence	Positions	Reference genome
**Puffin papillomaviruses**			
ATPU-Pap_F1	CTAGCCGCAATAATAATGGG	5978–5997	MK620302
ATPU-Pap_F2	CGCATAAATACCGCTATGGA	6007–6026	
ATPU-Pap_R1	AGTCGGGATACTTCGACACG	6255–6274	
**Gull and kittiwake papillomaviruses**			
HERG-Pap_F1	TAAACGCAGGCGTCTTCACC	5511–5530	MK620304
HERG-Pap_F2	TTTTACAGATGAGCGGCGCG	5536–5555	
HERG-Pap_R1	TGTTAGGAGACGGTCCGTAC	5653–5672	
**Duck papillomaviruses**			
MALL-Pap-F1	AGCCTGGTGTAGTGGTACAG	5715–5734	MK620303
MALL-Pap-F2	CTCTATTGCTGTGGTGGTTCC	5766–5786	
MALL-Pap-R1	GCATTGACCTTCGGTACAGC	6013–6030	

### Phylogenetic and Sequence Analyses

Viral sequences were visualized and assembled in Geneious R11 (Biomatters), which was also used for ORF prediction and genomic annotations. Identification of splicing acceptor sites was performed with NNSPLICE 0.9 (Reese et al., 1997). Predicted protein sequences were obtained by translating nucleotide sequences and protein domains typical of PVs were predicted by domain-search in Geneious using the Papillomavirus Episteme (PaVE) database^1^ as reference (Van Doorslaer et al., 2017a).

The complete genomic sequences and all individual proteins from all available complete APV genomes were downloaded from the GenBank database and used for protein comparisons and phylogenetic analyses. Partial sequences of APVs whose complete genomes were not available were also included and sequences from viruses of reptiles (*Caretta caretta* PV-1 and *Chelonia mydas* PV-1) and mammals (*Rousettus aegyptiacus* PV-1 and *Equus ferus caballus* PV-4) were used as an outgroup in phylogenetic analyses (accession numbers: NC_011530, EU493091, DQ366842, and JQ031032). Accession numbers of APV sequences used in this study are available in Supplementary Material (Supplementary Data File, APV features). Sequence alignments were performed with Clustal W (Larkin et al., 2007), manually edited if required, and maximum-likelihood (Felsenstein, 1981) phylogenetic trees were built with MEGA 7 (Kumar et al., 2016), using the best model for sequence distance estimation as predicted by model test analyses. Node robustness was evaluated with the bootstrap method (1000 replicates) (Felsenstein, 1985).

### Statistical Analyses

All statistical analyses on viral estimated prevalence (percentage of virus-positive individuals over the total number of individuals) were performed using R 3.1.2 (R Development Core Team, 2011). Positivity rates were analyzed separately for each virus and comparisons among groups were performed using a generalized linear model with a binomial distribution and a categorical variable. Variables considered included sampling locations (for Atlantic puffin only), species (for ducks and gulls), age (for ducks and gulls), sex (for ducks), and seasonality (for ducks).

### Data Availability

All sequences obtained in this study have been deposited in GenBank database under accession numbers MK620302-MK620305 for complete genomes and MK620306-MK620341 for partial sequences.

## Results

With the ViDiT-CACTUS virus discovery method several genomic fragments belonging to PVs were identified in various samples collected from wild birds (Verhoeven et al., 2018). Our genome-walking efforts allowed us to obtain the complete genomic sequences of four novel APVs, the puffin PV-1 (PuPV-1), the gull PV-1 (GuPV-1), the DuPV-3, and the kittiwake PV-2 (KiPV-2), which were identified in an Atlantic puffin, an American herring gull, a mallard and a black-legged kittiwake, respectively. For molecular-epidemiological investigations the complete L1 sequence was obtained from an additional 23 viruses and extended L1 sequences from another 14 viruses.

### Genome Organization of the Novel APVs

**FIGURE 1:**
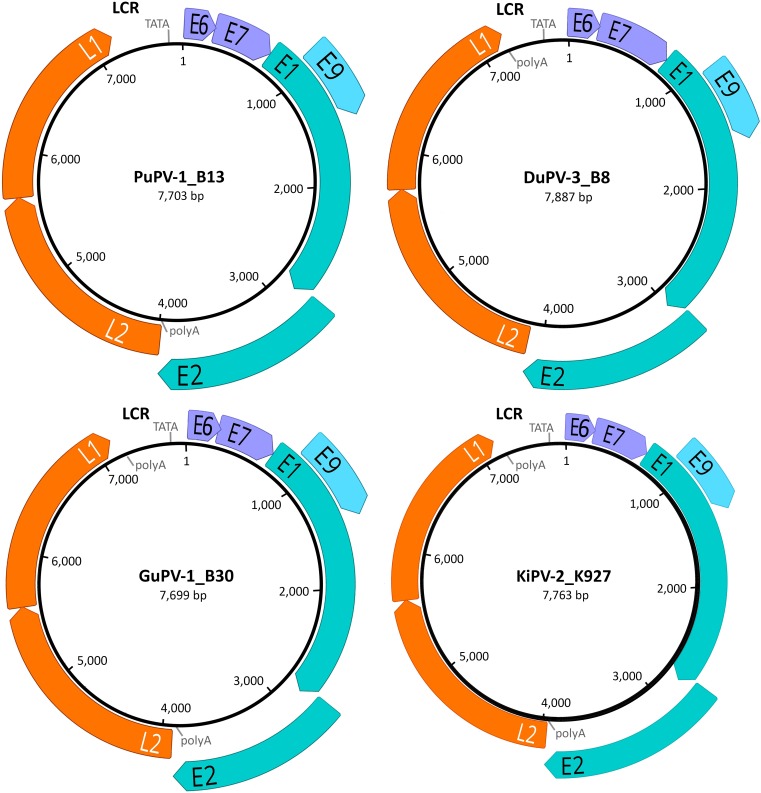
Genome organization of the four novel papillomaviruses identified in this study. For each virus (PuPV-1, puffin papillomavirus type 1; DuPV-3, duck papillomavirus type 3; GuPV-1, gull papillomavirus type 1, KiPV-2, kittiwake papillomavirus type 2), the size of the genome is indicated at the center of the diagram and predicted ORFs are represented by colored arrows (orange: late structural proteins; teal: core early non-structural proteins; purple: early accessory oncoproteins; light blue: avian-virus-specific putative early protein). The positions of the long control region (LCR), TATA box (TATA), and poly-adenylation signals (polyA) are also indicated.

The complete circular genomes of the four novel viruses were similar in size (approximately 7.7-7.9 kb) to other previously described APVs, which range between 7.3 and 8.1 kb. All viruses presented the typical PV genome organization with all four core ORFs coding for the proteins L1, L2, E1, E2, the ORFs coding for the E6 and E7 proteins, present in most PVs, and the additional ORF coding for the putative E9 protein, identified exclusively in avian viruses (all but FgPV-1) (Figure 1). The four viruses possessed an LCR region between the L1 and E6 ORFs and a short non-coding region between the E2 and the L2 ORFs.

Intriguingly, in all sequenced PuPV-1 strains, the predicted start codon for the L1 ORF was not a typical ATG but was substituted by GTG. Like in other PVs, this putative start codon overlaps the stop codon of the L2 ORF and is positioned immediately downstream from a highly supported splicing acceptor site (NNSPLICE score: 0.97). This, and the fact that the putative L1 protein generated from the GTG start codon is homologous to other APV L1 proteins, leads us to speculate that this GTG could be used as the start codon. No other similar instances were observed in other APVs.

### Molecular Features of APV Proteins and Non-coding Regions

In the following paragraphs we briefly describe the results of the comparisons of the molecular features among the viruses we discovered and all the other APVs. These findings are also summarized in the Supplementary Material (Supplementary Data File, APV features).

The LCR of PVs contains a series of elements that govern gene expression and replication, such as transcription factor and E1- and E2-binding sites (O’Connor et al., 1995). Among all investigated APVs, only three contained the typical E2-binding site (E2BS; ACCNNNNNNGGT), but high copy numbers (up to nine) of the atypical E2BS (ACCNNNNGGT) (Tachezy et al., 2002) could be identified in many more viruses, including DuPV-1 (*N* = 8), PuPV-1 (*N* = 2), GuPVs (*N* = 5–6), and the KiPVs (*N* = 8–9). Furthermore, most viruses contained the regulatory TATA box (TATAWAW) within the E6 gene promoter and a polyadenylation site (ATTAAA or AATAAA) for the mRNAs of the late genes (O’Connor et al., 1995). Another short (2–50 nt) non-coding sequence was present in most viruses between the E2 and L2 ORFs and this contained in some cases (PaPV-1, PuPV-1, GuPV-1, and KiPV-2) the predicted polyadenylation site for the late mRNAs.

**FIGURE 2:**
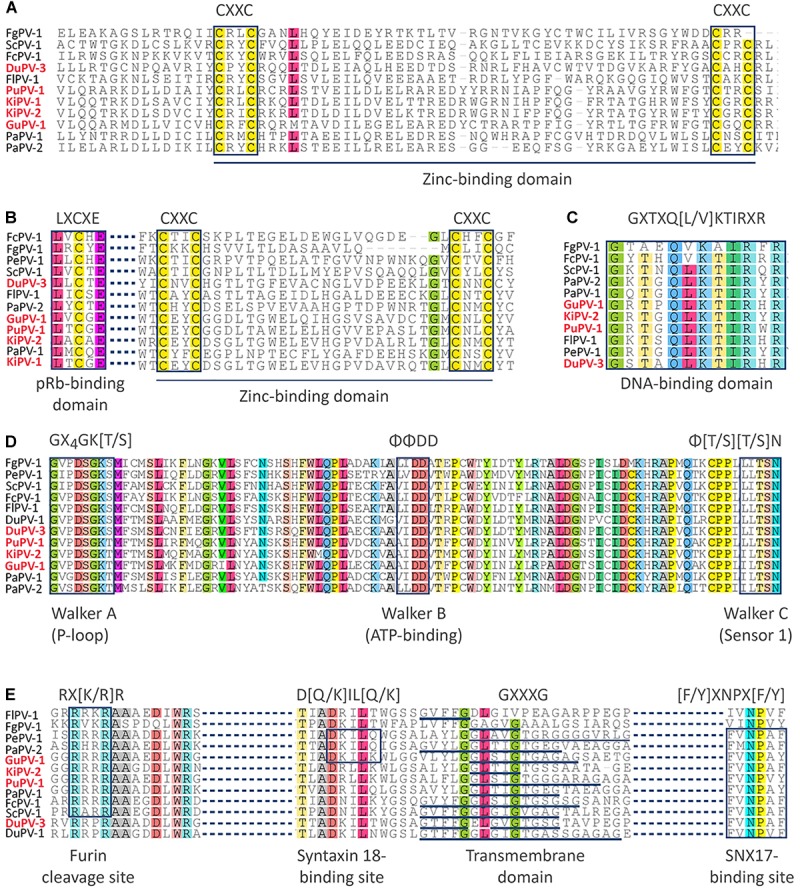
Alignments of conserved domains identified in APV proteins. The motifs of the zinc-binding domain are indicated in the sequences of the E6 protein **(A)** and E7 protein **(B)**, where the retinoblastoma tumor suppressor (pRb)-binding domain (LXCXE) is also specified. The DNA-binding domain of the E2 protein is shown in **(C)**, while the Walker domains of the E1 helicase are show in **(D)**. Finally, specific protein domains of the L2 protein are shown in **(E)**. Domain designation and typical sequences of each motif are indicated below and above the alignments, respectively. Viral types identified in this study are in red.

With the exception of PePV-1, which lacks the E6 ORF, all other viruses possessed both ORFs coding for the oncoproteins E6 and E7, which are responsible for the development of HPV-induced carcinogenesis (Tomaić, 2016). E6 proteins of APVs are 81–99 amino acids long, somewhat shorter compared to the E6 proteins of other PVs (approximately 150 amino acids long), and contain one unique predicted zinc-binding domain instead of two. This domain, formed by two pairs of CXXC motifs separated by 39–42 amino acids, was highly conserved across viruses, with the exception of FgPV-1 (Figure 2A). The N-terminal portion of the E7 protein of APVs is extended when compared to mammalian PVs (Van Doorslaer et al., 2009, 2017a). However, the typical retinoblastoma tumor suppressor (pRb)-binding domain (LXCXE) was present in the E7 of all APVs, although its location was variable among the different viruses, and the typical zinc-binding domain was present at the C-terminal side of all APV E7 proteins (Figure 2B).

The hexameric DNA helicase E1 is the only virus-encoded enzyme and is the most conserved PV protein (Bergvall et al., 2013). The E1 proteins of APVs ranged between 587 and 722 aa in length, somewhat larger than the E1 proteins from mammal-infecting viruses (600–650 aa). We were not able to identify the typical protein-localization signals within the N-terminal regulatory region of E1, probably because of high sequence divergence. However, the Walker A (phosphate-binding loop: GXXXXGK[T/S]), Walker B (ATP-binding domain: ΦΦDD), and Walker C (sensor 1: Φ[T/S][T/S]N) motifs that are characteristic of AAA+ (ATPases associated with various cellular activities) proteins were highly conserved within APV helicase domains (GXXDSGK[T/S], ΦΦDD, and ΦTSN, Figure 2D). Similarly, the DNA-binding domain could be identified within the C-terminal region of the E2 protein, which is an RNA transcription and viral replication regulator (McBride, 2013). This sequence (GXTXQ[L/V]KTIRXR) was highly conserved among all viruses, except FgPV-1 (Figure 2C).

Finally, several distinctive motifs could be identified that were conserved within the L2 structural protein among all APVs (Figure 2E). These included the furin cleavage motif (RX[K/R]R) in the N-terminal region of the protein that was found in all but the two duck viruses (DPV-1 and DuPV-3), a variable number of transmembrane GXXXG domains, and a sorting nexin 17 (SNX17)-binding site ([F/Y]XNPX[F/Y], found in all but FlPV-1 and FgPV-1. The furin cleavage site is essential for viral entry while the other two motifs are thought to be involved in endosomal escape (Wang and Roden, 2013). However, a syntaxin 18-binding site (D[Q/K]IL[Q/K]), involved in the transportation of the virus toward the nucleus (Wang and Roden, 2013), was only identified in PePV-1, PaPV-2, and GuPV-1.

### APV Estimated Prevalence, Diversity, and Distribution

To evaluate the distribution of the discovered viruses in local wild bird populations and to assess whether those viruses were species-specific or could infect closely related hosts, samples from species within the three bird families Alcidae, Laridae, and Anatidae were screened for PuPV-1, GuPV-1, and DuPV-3, respectively. Based on screening results we calculated the estimated prevalence of these viruses and the results are summarized in Table 2.

**Table 2:** Estimated prevalence of novel avian papillomaviruses in tested bird species.

Family	Species	Individuals	Positives (%)
**Alcidae^∗^**			
	Atlantic puffin, Witless Bay	9	2 (22.2)
	Atlantic puffin, Gannet Islands	42	3 (7.1)
	Atlantic puffin, total	51	5 (9.8)
	Common murre	41	0
	Thick-billed murre	2	0
	Razorbill	30	0
**Laridae**			
	American herring gull	94	16 (17.0)
	Great black-backed gull	38	1 (2.6)
	Iceland gull	4	0
	Ring-billed gull	9	0
	Black-legged kittiwake	16	13 (81.3)
**Anatidae**			
	Mallard	10	1 (10.0)
	American black duck	102	30 (29.4)
	American black duck × mallard	4	1 (25)

*^∗^Sampling sites are only indicated for PV-positive birds.*

All positive samples were confirmed by sequencing and, following PV classification rules (Van Doorslaer et al., 2017a), we assigned viruses to the same “type” when they shared >90% nucleotide sequence identity in the L1 ORF. Furthermore, since the whole genomic sequence necessary to define “variants” could not be obtained for all strains, we defined viruses that share >99% nucleotide sequence identity in the L1 ORF as “subtypes.” Typing results are summarized in Table 3.

**Table 3:** Putative types and subtypes of papillomaviruses identified in this study.

Type	Subtype	Host^a^	Accession number	Frequency (%)	Within-subtype identity (%)	Polymorphisms (NS)^b^
PuPV-1	1	ATPU	MK620302	6/6 (100)	99.5–100	5 (0)
DuPV-3	1	ABDU, MALL	MK620303	29/32 (90.6)^c^	100	0
	2	ABDU	MK620318	3/32 (9.4)^c^	100	0
GuPV-1	1	HERG	MK620304	9/17 (52.9)	99.8–100	4 (1)
GuPV-2	1	HERG	MK620329	6/17 (35.3)	100	0
	2	GBBG	MK620330	1/17 (5.9)	/	/
GuPV-3	1	HERG	MK620331	1/17 (5.9)	/	/
KiPV-1	1	BLKI	MK620332	2/13 (15.4)	100	0
KiPV-2	1	BLKI	MK620305	3/3 (23.1)	NA^d^	NA^d^
KiPV-3	1	BLKI	MK620334	1/13 (7.7)	/	/
KiPV-4^e^	1	BLKI	MK620336	4/13 (30.8)	100	100
KiPV-5^e^	1	BLKI	MK620339	1/13 (7.7)	/	/
KiPV-6	1	BLKI	MK620340	1/13 (7.7)	/	/
KiPV-7^e^	1	BLKI	MK620341	1/13 (7.7)	/	/

*^a^ATPU, Atlantic puffin; ABDU, American black duck; MALL, mallard; HERG, American herring gull; GBBG, great black-backed gull; BLKI, black-legged kittiwake. ^b^NS, non-synonymous substitutions. ^c^Subtype 1 was significantly more common than subtype 2 (df = 1, χ^2^ = 42.2, p < 0.001). ^d^NA, not available as very short sequences were obtained for two of the three strains. ^e^Partially sequenced.*

#### Papillomaviruses of Ducks

**FIGURE 3:**
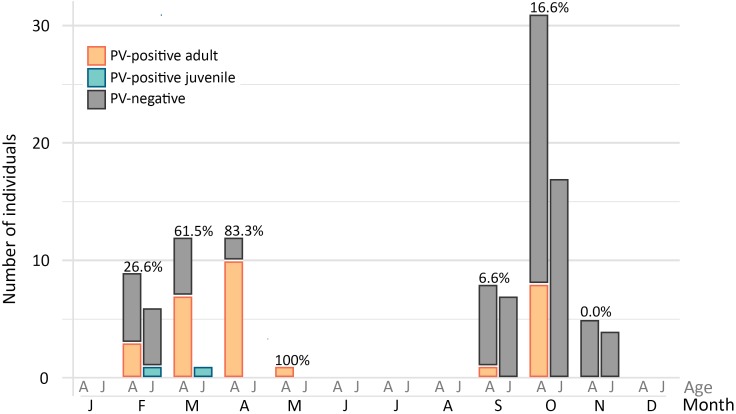
Estimated prevalence of DuPV-3 infection over the year. The graph illustrates the number of positive (colors) and negative (gray) adult (A) and juvenile (J) ducks (American black duck, mallard, and hybrids) sampled in each month of the year. Overall viral estimated prevalence for each month for which samples were available is indicated above the bars.

Duck papillomavirus-3 was originally identified in a mallard sampled within the city of St John’s, and the same virus was subsequently identified in samples collected from American black duck (29.4%) and also in one American black duck × mallard hybrid (Table 2). The estimated prevalence did not differ significantly by species (*df* = 2, χ^2^ = 1.70, *p* = 0.42). Among all ducks, estimated prevalence was significantly higher in adults (40%) than juveniles (5.6%) (*df* = 1, χ^2^ = 15.41, *p* < 0.001) but there was no difference between males and females (*df* = 1, χ^2^ = 1.66, *p* = 0.20). When accounting for differences between adults and juveniles, there was a strong seasonal pattern with high estimated prevalence in late winter/early spring (February to May: 23/41, 56.1%) and low estimated prevalence in the fall (September to November: 9/72, 12.5%) (*df* = 1, χ^2^ = 24.2, *p* < 0.001) (Figure 3). Finally, positive individuals were identified at all three different locations within the city of St. John’s at similar rates (39.5–50%) and, among the 32 positive individuals, we observed two distinct subtypes (Table 3). Over the whole L1 sequence, the two subtypes were 95.7% identical to each other and 66 nt substitutions, all synonymous, differentiated the two strains. For three DuPV-3-positive birds sampled in 2018 the cloacal and the oropharyngeal swabs were preserved separately and, in all cases, only the oropharyngeal swab was PV-positive. Finally, an archived sample that was collected from one positive American black duck 1 year earlier was negative for the virus.

#### Papillomaviruses of Puffins

Puffin papillomavirus-1 was originally identified in a sample collected from a puffin in the Witless Bay Ecological Reserve. In total, we identified five positive puffins (estimated prevalence: 9.8%), two from Witless Bay, and three from the Gannet Islands Ecological Reserve. All sequences were 99.5–100% identical (Table 3). No geographic segregation was observed, and viral estimated prevalence did not differ between the two locations (*df* = 1, χ^2^ = 1.56, *p* = 0.21). All samples were collected in July and August, during the breeding season, and all came from breeding adults. Interestingly, an archived sample collected from one of the positive puffins approximately 1 year earlier was also positive and the viral sequences obtained from the two samples from this individual were 100% identical. Finally, no other sample from other bird species within the family Alcidae (thick-billed and common murre and razorbill) was found positive for this virus in either the Witless Bay or Gannet Islands Reserves (Table 2).

#### Papillomaviruses of Gulls

Gull papillomavirus-1 was originally identified in an American herring gull sampled in the city of St. John’s. Total PV estimated prevalence within the herring gull population was 17%, one great black-backed gull was positive (2.6% estimated prevalence), but PVs were not found in the small numbers of Iceland or ring-billed gull samples screened. PV presence was significantly higher in herring gull compared to the other gull species (*df* = 3, χ^2^ = 9.79, *p* = 0.02). Although it did not reach statistical significance, a higher viral presence was identified in juvenile herring gull individuals (24.1% vs. 13.8%; *df* = 1, χ^2^ = 1.43, *p* = 0.23). Genetic diversity was high and three different viral types were identified (GuPV-1–GuPV-3, Table 3). Over the whole L1 sequence GuPV-2 showed the greatest diversity, with two subtypes that were 98.1% identical to each other and differed by 30 nucleotide substitutions, eight of which were non-synonymous. Interestingly, one subtype was observed in the great black-backed gull and the other subtype in herring gull.

#### Papillomaviruses of Kittiwakes

A very high PV estimated prevalence (81.3%) was detected in black-legged kittiwake, and sequence analysis showed that the viral types circulating in this species were different from those present in the other gulls. All sampled individuals were adults and samples were all collected in July 2015 at the same location. Among the 13 positive birds we identified a very high viral diversity, with seven different types (KiPV-1–KiPV-7, Table 3). Unfortunately, for three of the identified types we only obtained about 85% (KiPV-4 and KiPV-7) or 55% (KiPV-5) of the L1 ORF sequence.

### Phylogenetic Analysis of APVs

**FIGURE 4:**
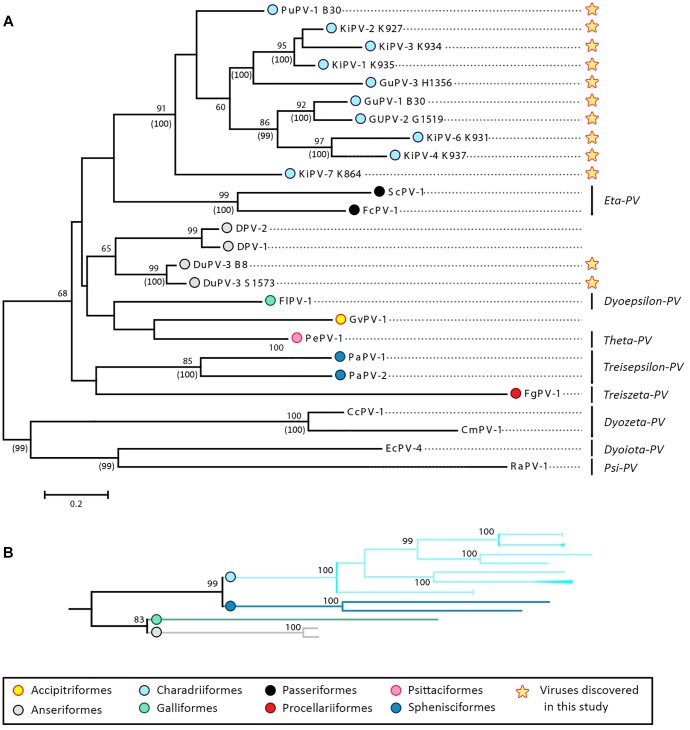
Phylogenetic analysis of APVs. **(A)** Analysis of partial L1 nucleotide sequences (corresponding to nt 5678–6070 of FcPV-1; accession number NC_004068) of all known APVs. The tree was built with the maximum-likelihood method (Felsenstein, 1981) based on the Kimura 2 parameters model (Kimura, 1980), identified as the best-fitting model after the model test analysis in MEGA 7 (Kumar et al., 2016). A discrete Gamma distribution was used to model evolutionary rate differences among sites (+*G* = 0.9136) and branch lengths are proportional to genetic distances as indicated by the scale bar. The outcome of the bootstrap analysis (Felsenstein, 1985) is shown next to the nodes, while the corresponding bootstrap values obtained in a different analysis involving the whole L1 open-reading frame (Supplementary Figure S1) is shown in parenthesis. Viruses are marked by type name and the strain name is also indicated for viruses described in this study (indicated by a star), while established viral genera are shown on the right. Viruses are labeled by a colored dot that indicates the order of the birds in which they were identified, as indicated in the legend, and hosts are indicated within viral type names (Du, duck; Fc, *Fringilla coelebs*; Fg, *Fulmarus glacialis*; Fl, *Francolinus leucoscepus*; Gf, *Gyps fulvus*; Gu, gull; Ki, kittiwake; Pa, *Pygoscelis adeliae*; Pe, *Psittacus erithacus timneh*; Pu, puffin; Sc, *Serinus canaria*; CC, *Caretta caretta*; Cm, *Chelonia mydas*, Ec, *Equus ferus caballus*, Ra, *Rousettus aegyptiacus*). **(B)** Subtree including branches of viruses identified in four bird orders (Charadriiformes, Sphenisciformes, Galliformes, and Anseriformes) obtained from the phylogenetic analysis involving the complete L1 ORF (Supplementary Figure S1).

Figure 4A shows the phylogenetic relationships among currently known APVs and their relationships to representative strains from reptiles and mammals. To achieve the most complete representation possible and consider all available types and subtypes, including those for which only partial sequences are available, we based this tree on partial L1 sequences (approximately 430 nt). However, a tree based on complete L1 sequences from a subset of strains is available in the Supplementary Material (Supplementary Figure S1).

The phylogenetic analyses revealed a monophyletic origin of APVs, with all bird viruses included in a clade that was separated from other vertebrate viruses (Figure 4A and Supplementary Figure S1). Our analyses also provide evidence for virus–host co-evolution. Viruses infecting birds belonging to the same family formed distinct, supported, and monophyletic clades, as seen for the two Adélie penguin (PaPV-1 and -2) and the three duck (DuPV-1, -2, and -3) viruses. Furthermore, viruses that infect birds within the same order also formed bootstrap-supported monophyletic groups, and this was especially evident for viruses infecting birds within the orders Passeriformes (FcPV-1 and ScPV-1) and Charadriiformes (PuPV-1, GuPV-1, -2, and 3, and KiPV-1–KiPV-7). Additionally, as illustrated in Figure 4B, the tree based on the whole L1 showed support for a clade that includes viruses infecting Galliformes (FlPV-1) and Anseriformes (DuPV-3), reflecting the closer phylogenetic relationship between those two orders (Prum et al., 2015). The same was observed for the orders Charadriiformes and Sphenisciformes, both members of the clade Aequorlitornithes, whose close phylogenetic relationship was well matched by the highly supported clade of viruses that included all those from Charadriiformes and the two Adélie penguin viruses.

**FIGURE 5:**
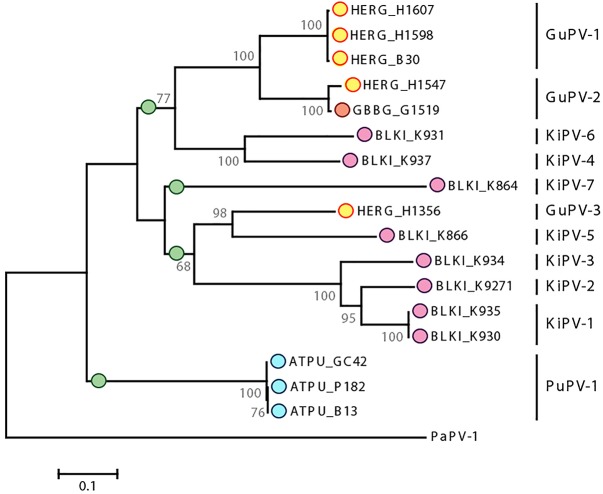
Phylogenetic analysis of APVs identified in birds within the order Charadriiformes. The tree is based on L1 sequences of all PVs identified in this study from Atlantic puffin (ATPU, blue), American herring gull (HERG, yellow), great black-backed gull (GBBG, orange), and black-legged kittiwake (BLKI, pink). The four proposed different species are indicated by green dots at the base of the species branch. The tree was built with the maximum-likelihood method (Felsenstein, 1981) based on the Tamura–Nei model (Tamura and Nei, 1993), identified as the best-fitting model after the model test analysis in MEGA 7 (Kumar et al., 2016). A discrete Gamma distribution was used to model evolutionary rate differences among sites (+*G* = 0.9236) and the rate variation model allowed for some sites to be evolutionarily invariable ([+I], 23.03% of sites). Branch lengths are proportional to genetic distances as indicated by the scale bar and the outcome of the bootstrap analysis (Felsenstein, 1985) is shown next to the nodes. Viral types or putative types are indicated on the right, and PaPV-1 was used as an outgroup.

Our analyses also indicate that host specificity constrains are relaxed among viruses infecting phylogenetically close birds. A phylogenetic analysis focused on viruses of the Charadriiformes and performed with a longer L1 fragment (approximately 600 nt) shows the presence of several highly supported clades, but the viral clusters do not always correspond to the relationships among the infected bird species (Figure 5), highlighting possible past and ongoing host-switching events. Specifically, the gull viruses GuPV-1 and GuPV-2 show a closer relationship to KiPV-4 and KiPV-6, rather than to GuPV-3, which is more closely related to KiPV-5 and the KiPV-1, -2, and -3 clade (Figure 5).

### Taxonomic Implications

The International Committee for the Taxonomy of Viruses (ICTV) criteria for PV classification are based on a combination of nucleotide identity cut-offs for the L1 ORF and the L1 phylogeny. The demarcation between phylogenetically supported taxa is set at 60% for genera, 70% for species, and 90% for types (de Villiers et al., 2004; Van Doorslaer et al., 2017a, 2018). The average pairwise identities between the L1 nucleotide sequences of each APV type (expressed as the inverse of the mean *p*-distance between groups) were calculated and is shown in the Supplementary Data File (APV genetic identities). The L1 of DuPV-1 and DuPV-2 are 91% identical and these viruses could be classified as the same viral type, while all DuPVs could belong to the same genus and, likely, to the same species since the average pairwise identity between them is approximately 74%. Furthermore, it is possible that all DuPVs could be classified within the genus *Dyoepsilonpapillomavirus* (identity between DuPV-1, -2, and -3 and FlPV-1: 62.4–65.9%), although the existence of this clade was only supported when the whole L1 sequence was used for phylogenetic reconstruction (Figure 4B and Supplementary Figure S1).

Table 4 shows the average pairwise identities between viral types found in Charadriiformes. GuPV-1 and GuPV-2 meet the criteria to be two different types within the same species (approximately 84% pairwise identity), and this species also potentially includes the kittiwake viruses KiPV-4 and Ki-PV6, as pairwise identities between gull and kittiwake viruses within this clade are >70%. The three kittiwake viruses KiPV-1, KiPV-2, and KiPV-3 can be classified within the same species (average pairwise identities approximately 85–88%) and GuPV-3 and KiPV-5 potentially belong to the same species (pairwise identity of approximately 73%). However, it is possible that these five viruses all belong to the same species as identity between GuPV-3 and the clade KIPV-1–KIPV-3 is >70% and KiPV-5 has only been partially sequenced. Finally, the other two charadriiform viruses (PuPV-1, KiPV-7) likely represent two distinct species. These four distinct proposed species of viruses from the Charadriiformes, indicated by green dots in Figure 5, are similarly distant from each other, with an average between-species identity of 65–70%, and they all likely belong to the same novel genus.

**Table 4:** Average pairwise nucleotide identities (percentage of 1-*p* distance) between APV types and putative types identified in Charadriiformes^a^.

	GuPV-1	GuPV-2	KiPV-4^b^	KiPV-6	KiPV-5^c^	GuPV-3	KiPV-1	KiPV-2	KiPV-3	KiPV-7^b^
**GuPV-2**	**84.1**									
**KiPV-4^b^**	**74.9**	**74.8**								
**KiPV-6**	**72.0**	**72.1**	**80.0**							
**KiPV-5^c^**	64.7	66.1	69.3	64.6						
**GuPV-3**	67.8	69.5	70.7	68.7	**72.7**					
**KiPV-1**	68.9	68.6	70.1	67.6	**66.5**	**73.7**				
**KiPV-2**	68.3	69.6	70.5	66.9	**68.6**	**73.4**	**88.8**			
**KiPV-3**	68.4	69.1	69.9	68.3	**68.4**	**72.0**	**85.0**	**85.4**		
**KiPV-7^b^**	67.6	68.4	68.3	67.3	67.9	68.6	68.2	68.5	68.1	
**PuPV-1**	69.7	70.5	71.2	69.2	63.4	68.8	69.1	69.2	68.5	67.2

*^a^Groups of identities between viruses potentially belonging to the same species are in bold. ^b^Approximately 85% of the L1 gene. ^c^Approximately 55% of the L1 gene.*

## Discussion

With over 10,000 living species, birds are the most diverse extant lineage of tetrapod vertebrates (Prum et al., 2015). They have a global distribution, are known to harbor numerous microbes, and are important reservoir hosts for many pathogenic bacteria and viruses (Olsen et al., 2006; Ayginin et al., 2018; Chung et al., 2018). Furthermore, many birds migrate over broad spatial scales and across biogeographical borders and therefore can have important roles in the long-distance dispersal of microbes and contribute to defining the ecology and shaping the evolution of different viruses and bacteria (Viana et al., 2016).

Although the diversity and distribution of several specific viruses within birds have been investigated to varying degrees (Rappole et al., 2000; Olsen et al., 2006; Chamings et al., 2018; Jardine et al., 2018), our knowledge about overall viral diversity in the avian reservoir is still very limited, which is highlighted by the continuously increasing number of viruses discovered in these hosts (Chan et al., 2015; Bodewes, 2018). Among these, PVs are particularly interesting since only eight different viral types have been described so far in seven bird species, but there are likely many more to be discovered. In this study we describe molecular, epidemiological, and evolutionary features of several novel APV types we discovered in wild birds with the ViDiT-CACTUS method (Verhoeven et al., 2018).

### Molecular Features of the Novel APVs

For four of the viruses we discovered (DuPV-3, GuPV-1, PuPV-1, and KiPV-2) we were able to obtain the complete genomic sequences and we showed that they have the same genome organization identified in other APVs, including the presence of the bird virus-specific E9 ORF nested within the E1 ORF (Van Doorslaer et al., 2017b). Furthermore, we observed other genetic traits that are typical of APVs, such as the presence of only one zinc-binding domain within the E6 protein, an extended E7 protein, and the presence of an atypical E2BS within the LCR (Tachezy et al., 2002; Van Doorslaer et al., 2009, 2017b). Finally, although the search for specific PV domains was made difficult by the low sequence identity between avian and non-avian PVs, some domains were highly conserved among APV proteins and these included the pRb-binding domain within E7 (Tomaić, 2016), the Walker domains typical of the E1 helicase (Bergvall et al., 2013), the DNA-binding domain in E2 (McBride, 2013), and the furin cleavage site and SNX17-binding site in L2 (Wang and Roden, 2013).

Intriguingly, the L1 ORF of all three sequenced strains of PuPV-1 contained a GTG codon at the predicted L1 start codon position. This prediction is based on other APVs where the L1 start codon overlaps the stop codon of the L2 ORF and is immediately downstream of a highly supported splicing acceptor site. Furthermore, the predicted L1 protein, when translated as starting from this GTG codon, is homologous to other APV L1 proteins. Although yet to be experimentally substantiated, these genetic features lead us to hypothesize that this GTG may be used as an alternative start codon in the puffin viruses, as also previously hypothesized for an avian circovirus (Todd et al., 2007). GTG is frequently used by bacteria as a start codon (Villegas and Kropinski, 2008) and there is evidence for the use of GTG as an alternative start codon in mitochondria (Bock et al., 1994; Perseke et al., 2011). Furthermore, it has been demonstrated that GTG can be used as an alternative start codon in *Drosophila melanogaster* (Sugihara et al., 1990) and there is *in vitro* experimental evidence for its use to start protein translation in mammalian cell lines (Fuxe et al., 2000; Hwang et al., 2005). Use of this alternative start codon has been shown to lead to lower protein production in comparison to ATG (Sugihara et al., 1990; Hwang et al., 2005), so using this codon may be a way for the virus to regulate the expression of the L1 protein. However, pending experimental validation, this remains speculation and it is also possible that the virus uses an ATG start codon that is located at an atypical position for L1 translation initiation.

### Molecular Epidemiology of APVs in the Anatinae

Including those found in this study, four different PVs have now been described in ducks [tribe Anatini within the sub-family Anatinae (Gonzalez et al., 2009)]. The L1 nucleotide sequences of DuPV-1 (Fawaz et al., 2016) and DuPV-2 (Williams et al., 2018) are approximately 92% identical, but only a partial sequence is available and it is not currently possible to evaluate whether they represent two different viral types or two variants of the same type. The two viruses we identified in this study represent two subtypes of the same type (DuPV-3) and, according to the preliminary sequencing data, all three duck viruses belong to the same viral species, possibly within the genus *Dyoepsilonpapillomavirus*. Within this genus, all DuPV types are monophyletic, suggesting a common ancestor.

Although epidemiological information is still limited, there is evidence for a wide geographic distribution of DuPV-1 as the same virus was detected in India and Sweden. Interestingly, regardless of the geographic location where viruses were identified, all sequenced strains belonging to the same DuPV subtype are 100% identical and this is suggestive of a long association of the virus with its host. Furthermore, none of the 66 nucleotide substitutions identified between the two DuPV-3 subtypes caused an amino acid substitution and only approximately 6% of the nucleotide substitutions between DuPV-1 and DuPV-2 were non-synonymous, indicating strong negative selection pressure on the L1 capsid protein.

In terms of host specificity, DuPV-1 was found in *A. platyrhynchos* and *A. platyrhynchos domestica*, DuPV-2 in *A. platyrhynchos domestica*, and DuPV-3 in *A. platyrhynchos*, *A. rubripes*, and their hybrids. All of these species are considered part of a mallard-type complex and whether they even represent good species has been questioned (Mank et al., 2004). Any genetic barriers are, therefore, probably small and easy for viruses to bypass. Furthermore, there are no ecological barriers for virus transmission because they often live in close proximity in crowded environments and interbreed. These aspects of duck ecology favor viral transmission and, in turn, may influence viral ecology and evolution. Investigating PVs infecting other *Anas* spp. that are not part of the mallard-type complex (e.g., wigeons or teals) will be instructive in determining the importance of host species for cross-infectivity.

We observed a high estimated prevalence (approximately 30%) of DuPV-3 in the American black duck population and a lower circulation rate in mallard (approximately 10%) with evidence of seasonality in infections. These data are in agreement with previous reports, although infection rates previously documented were lower (1.6% and 12% in mallard and domestic ducks, respectively) (Williams et al., 2018). This difference may be partially explained by the fact that the study by Williams et al. (2018) considered only fecal or cloacal swab samples, while our study included paired oropharyngeal and cloacal swabs and we showed in the three cases where we had separated swab samples that DuPV-3 was only detected in the oropharyngeal samples.

Duck papillomavirus-3 was significantly more prevalent in adult ducks and all DuPV-1- and DuPV-2-positive ducks were adults. Williams et al. (2018) hypothesized a possible sexual route of viral transmission, but our data suggest other transmission routes are also likely. The higher infection rate found in adults and during the pre-breeding season could support a sexual route of transmission; however, viruses were also detected in juvenile birds and we showed the virus could be present in the oropharyngeal compartment and not in the cloaca. Finally, the occurrence of persistent infection was demonstrated for DuPV-1 and DuPV-2, but we could not prove whether this is the case for DuPV-3 as sequential samples were only available for one individual that did not show evidence of persistent infection. Although many pieces of the puzzle are still missing for all three duck viruses, and future studies may reveal more common traits, it is possible that DuPV-1 and -2 possess a different tropism than DuPV-3 and this would explain differences in viral ecology and distribution.

### Molecular Epidemiology of APVs in Charadriiformes

Within the order Charadriiformes, we found four bird species (American herring gull, great black-backed gull, black-legged kittiwake, and Atlantic puffin) in two bird families (Laridae and Alcidae) infected by PVs. Viruses identified in these birds all meet the criteria to belong to the same novel genus and included 11 different types and several subtypes. Only one viral type was identified in puffins, while a very high viral genetic diversity was observed within the Laridae, where 10 distinct viral types were identified among 30 individual birds, with the highest viral diversity observed in black-legged kittiwake (7 types among 13 infected individuals). Differently from what was observed for the duck viruses, we identified non-synonymous substitutions, indicating a weaker negative selection pressure on the L1 capsid protein, and suggestive of a more recent virus–host association. Unfortunately, only partial L1 sequences could be obtained for some of the types and obtaining the complete genomic sequences of more viral types is therefore a priority.

Among the Laridae we found evidence for cross-species infection as the same virus was found in American herring and great black-backed gull. Both species were sampled at the same time and place and it is possible that habitat sharing is facilitating cross-species transmissions. However, the large white-headed gulls (which include these two species) are a closely related genetic complex with evidence of recent introgression (Sonsthagen et al., 2016), possibly reducing barriers to cross-species transmission. Viral estimated prevalence was high in American herring gull (17%) and only one positive great black-backed gull was identified, pointing toward American herring gull being the main host for these viruses, although a smaller number of samples from great black-backed gull was tested. No cross-species transmission was identified for the other viruses.

A remarkably high estimated viral prevalence was observed in black-legged kittiwake (>80%) and this pattern could be explained by host-related factors. Kittiwakes nest on cliff ledges in colonies with individuals in close proximity (Hatch et al., 2009), behavior that could facilitate viral spread. On the contrary, puffins excavate nesting burrows into the soil, causing birds to be somewhat more segregated (Harris and Wanless, 2011), possibly explaining their lower estimated viral prevalence (approximately 10%). It should be noted, however, that puffins are very social, participating in various behavioral displays and regularly interacting outside their burrows (Harris and Wanless, 2011). An alternative explanation is that the primers used to screen samples collected from the Alcidae were more species-specific than those used to screen samples from the Laridae, resulting in an artificially lower estimate of prevalence. Finally, the chance that viral infection rates are related to sexual promiscuity is low as extra-pair copulations are rare in kittiwakes (Helfenstein et al., 2004), gulls (Gilbert et al., 1998), and puffins (Creelman and Storey, 1991).

Interestingly, puffins sampled in two distant locations (straight-line distance of approximately 500 km) shared similar viruses and this is possibly related to the migration patterns of these animals, which over-winter on the Grand Banks off the coast of Newfoundland (Fayet et al., 2017), facilitating viral mixing and dispersal. Kittiwakes similarly over-winter on the Grand Banks, with birds that breed in North America mixing with those from colonies in Europe (Bogdanova et al., 2011; Frederiksen et al., 2011), possibly explaining the co-occurrence of several different types. Gulls are also migratory (Nisbet et al., 2017) and this can provide further opportunities for viral mixing, as shown already for avian influenza virus (Wille et al., 2011a,b; Hall et al., 2013; Dusek et al., 2014; Huang et al., 2014).

Among the Charadriiformes, samples from both juveniles and adults were only available for gulls and interestingly, although not significantly, viral presence was higher in juvenile birds compared to adults, showing a different trend compared to PVs in ducks. However, additional screening will be required to confirm these data. Finally, we have evidence for a possible persistent infection in one puffin, although we cannot exclude the possibility that this individual cleared an initial infection and was subsequently re-infected with the same virus.

### Ecology and Evolution of APVs

In total, we identified 12 APV types, which meet the criteria to be potentially classified in five species and two genera, substantially expanding our knowledge about PV diversity in the avian reservoir. However, some of the analyzed L1 sequences were not complete and identities between types, used to evaluate demarcation between species and genera, will have to be reevaluated when more sequence information is available. Our results also increase the number of avian species known to harbor PVs from 7 to 12 and demonstrate the presence of PVs in an additional avian order, the Charadriiformes. However, considering the vast PV genotype diversity existing in other hosts and the number of currently living bird species, many more APVs likely exist. Therefore, our conclusions in terms of ecology and evolution of APVs are preliminary as they are based on a relatively limited number of viruses.

Our results substantiate current ideas about the evolution of PVs, where virus–host co-divergence dominates the evolutionary history of PVs and cross-species transmission events are sporadic and limited to genetically close hosts (Gottschling et al., 2007; Shah et al., 2010; Bravo and Félez-Sánchez, 2015). In our study APVs showed a monophyletic origin. However, the phylogenetic placement of FgPV-1 within the avian clade was not consistent across analyses and other studies already reported its position cannot be determined with certainty (Van Doorslaer et al., 2017b). We observed that different viral types (and species) infecting birds classified within the same order tend to cluster together, mirroring the phylogeny of their avian hosts. This was evident for all four viral genera containing more than one viral species where the two different species within the genus *Etapapillomavirus* infect two members of the order Passeriformes, the two viral species within the genus *Treisepsilonpapillomavirus* both infect Adélie penguin, all three duck viruses were grouped within the same genus, and all viruses found in birds within the Charadriiformes (both families Laridae and Alcidae) could be grouped within the same genus. Furthermore, with some exceptions, different viruses infecting one host species tend to have a monophyletic origin. We could also observe that the closer relationship between the two bird orders Anseriformes and Galliformes was reflected by the presence of a viral clade (possibly corresponding to the genus *Dyoepsilonpapillomavirus*) containing all viruses identified to-date in birds belonging to these taxa (the duck viruses and FlPV-1). Finally, the same was observed for viruses of the Charadriiformes and Sphenisciformes, whose close phylogenetic relationships match that of their hosts. In this scenario, the relationship of the virus identified in the Northern Fulmar (FgPV-1) to other APVs is puzzling. Its unusual genomic organization, as well as its uncertain phylogenetic placement, may suggest the existence of two different major APV clades.

The opposite trend was observed when considering viruses infecting birds within the same order or family. In particular, viruses infecting birds within the Charadriiformes formed several independent and highly supported clades that did not coincide with the host species in which they were found. Specifically, we identified two highly supported clades including viruses found in birds from the genera *Larus* and *Rissa* and found that some gull viruses are more closely related to kittiwake viruses than to each other. These intricate connections demonstrate that a possibly extensive exchange of viruses has occurred among Laridae in the past, which allowed several viral lineages to diverge in related bird species. Furthermore, the gull viral-type GuPV-2 and the duck viral-type DuPV-3 were identified in two different host species (albeit closely related species that are able to hybridize), indicating that cross-species transmission still occurs and that host-specificity constraints are relaxed among more closely related hosts, as already reported for other non-avian PVs (Gottschling et al., 2007; Chen et al., 2009). However, the two GuPV-2 strains identified in American herring gull and great black-backed gull were only approximately 98% identical, possibly indicating a recent strain segregation. In this context, host ecology and behavior may be important factors in determining viral ecology, distribution, transmission patterns, and, eventually, evolution.

In conclusion, we describe the genetic and epidemiological traits of several novel APVs discovered in six different bird species, some of which were not previously known to harbor PVs. We demonstrate that the genome organization of all currently known APVs is very similar and that there is some synteny in the phylogenetic relationships of APVs and their avian hosts, with evidence for virus–host co-divergence. Cross-species transmission events seem to occur between genetically closely related hosts and our findings show how host behavior can affect viral ecology and evolution.

## Ethics Statement

This study was carried out in accordance with the recommendations of the Canadian Council on Animal Care. The protocol was approved by the Memorial University Institutional Animal Care Committee (approved protocols 11-01-AL, 12-01-AL, 13-01-AL, 14-01-AL, and 17-05-AL). Wild birds were captured under federal authority (Canadian Wildlife Service Migratory Bird Banding Permit 10559).

## Author Contributions

MC and AL conceived the study. MC, HM, GR, AK, SR, and HW collected the samples. MC, AK, SR, and DO performed the laboratory procedures. MC performed all sequence and phylogenetic analyses, and data and statistical analyses were performed by HM. MC wrote the manuscript with the contribution of HM and the assistance of GR and AL. All authors read and approved the final version of the manuscript.

## Conflict of Interest Statement

The authors declare that the research was conducted in the absence of any commercial or financial relationships that could be construed as a potential conflict of interest.

## Footnotes

1 pave.niaid.nih.gov
