# Supplementary material for: New Insight Into Avian Papillomavirus Ecology and Evolution From Characterization of Novel Wild Bird Papillomaviruses
Source: Front Microbiol. 2019 Apr 12;10:701. doi: 10.3389/fmicb.2019.00701 (PMC6473165; doi:10.3389/fmicb.2019.00701)

New insight into avian papillomavirus ecology and evolution from characterization of novel wild bird papillomaviruses

Marta Canuti, Hannah J. Munro, Gregory J. Robertson, Ashley N.K. Kroyer, Sheena Roul, Davor Ojkic, Hugh G. Whitney, Andrew S. Lang

Supplementary Figure S1. Phylogenetic analysis of avian papillomaviruses. The analysis is based on the full L1 nucleotide sequence of known avian papillomaviruses whose complete L1 sequences are available. The tree was built with the maximum-likelihood method based on the Kimura 2 parameters model, identified as the best fitting model after the model test analysis in MEGA 7 (Kumar, S., Stecher, G., and Tamura, K. (2016). MEGA7: Molecular Evolutionary Genetics Analysis version 7.0 for bigger datasets. *Mol Biol Evol* 33, 1870–1864. doi:10.1093/molbev/msw054). A discrete Gamma distribution was used to model evolutionary rate differences among sites (+G = 1.4655) and branch lengths are proportional to genetic distances as indicated by the scale bar. The outcome of the bootstrap analysis is shown next to the nodes. Viruses are marked by the type name and the strain name is also indicated for viruses described in this study. Viruses are labelled by a colored dot that indicates the order of the birds in which they were identified, as shown in the legend.

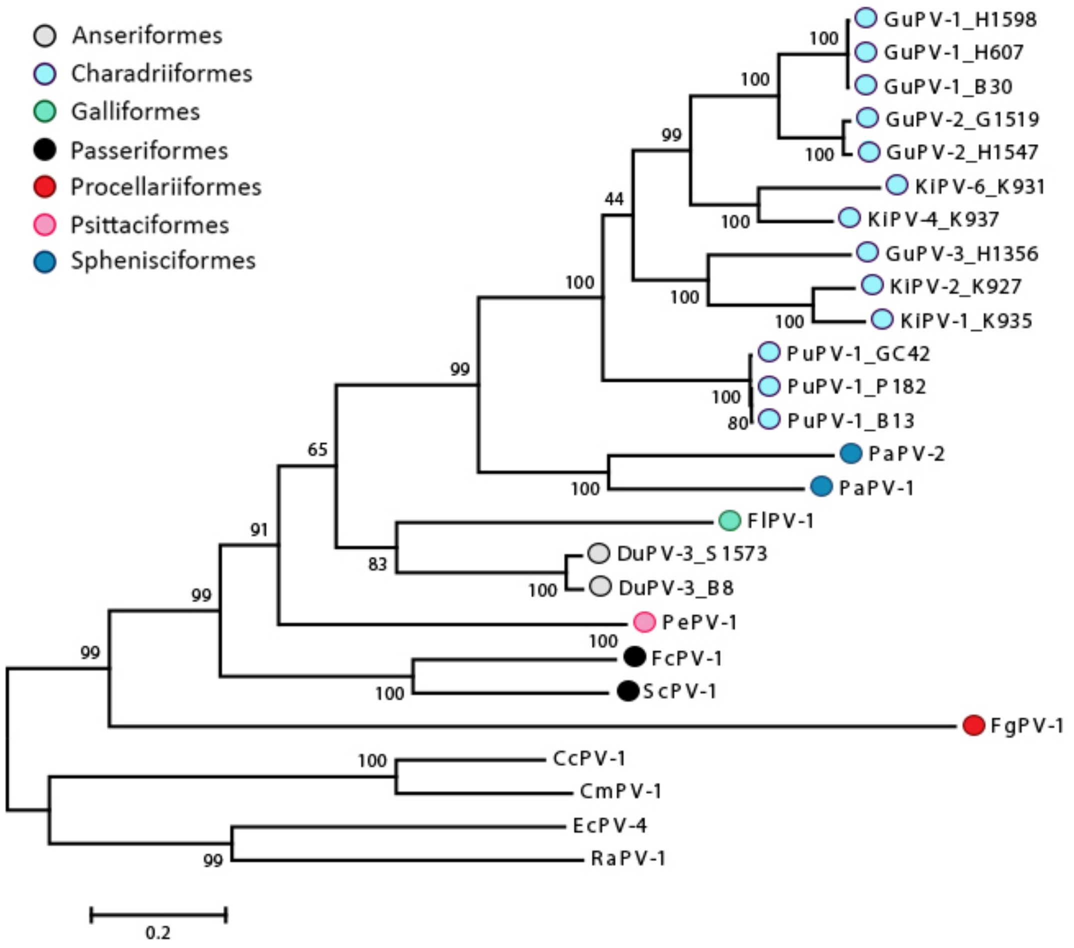

Supplement: Supplementary file 1 [file Data_Sheet_1.PDF]
